# Supplementary material for: Platelet Distribution Width Is Associated with P-Selectin Dependent Platelet Function: Results from the Moli-Family Cohort Study
Source: Cells. 2021 Oct 13;10(10):2737. doi: 10.3390/cells10102737 (PMC8535046; doi:10.3390/cells10102737)
Supplement: Supplementary file 1 [file cells-10-02737-s001.zip › cells-1412868-supplementary.pdf]

Platelet Distribution Width Is Associated with P-Selectin Dependent Platelet Function: Results from the Moli-Family Cohort Study

Supplementary Materials

Supplementary Methods

Study Population

The Moli-family cohort [1–3] includes 754 white subjects (≥ 15 years old) from 54 extended pedigrees (23 families with personal or familial history of early myocardial infarction (MI)—MI families—and 31 families without MI) recruited in Molise, a Southern Italy region. All participants were relatives of index subjects enrolled in the population-based Moli-sani study [4]. To be included in the cohort, a family had to have at least 10 living relatives over three generations. The index subject of each family with early MI was either a patient who had suffered an MI before 45 years or a patient with MI before 65 years who had at least a first degree relative with cardiovascular disease (CVD) before 65 years. The index subject of each family without CVD was a subject matched by age and sex with no personal or family history of CVD [5]. In all subjects, a complete medical history and information about smoking and alcohol-drinking habits were obtained via a structured questionnaire. Height, body weight, and blood pressure were measured as described [5]. Blood samples were obtained between 07:00 and 09:00 from participants who had fasted overnight and had refrained from smoking for at least 6 h. Enrollment and data collection were performed as previously described [5]. The Moli-family study was approved by the Ethical Committee of the Catholic University in Rome, Italy. All subjects provided written informed consent; adult subjects also provided informed consent for their minor children.

Flow Cytometry Measurements

Mixed platelet-leukocyte conjugates and markers of platelet activation were measured in the Moli-family participants as described [1]. Briefly, venous blood was collected in 3.8% trisodium citrate vacutainer tubes and processed between 10 and 20 min after collection. Whole blood was then either immediately fixed using a commercially available fixative (Thrombo-Fix™, Beckman Coulter Inc.) or previously stimulated *in vitro* using ADP and collagen. Details of the protocol were reported before [1]. Platelet-leukocyte conjugates and platelet P-selectin were measured in whole blood as described [1]. Platelets, including platelet aggregates, were defined by morphological characteristics and by CD42b positivity. Platelet-leukocyte aggregates were identified by counting the $CD61^+$ events among the polymorphonuclear (PMN) leukocyte and monocyte populations defined on the basis of the side scatter (SSc) characteristics within the $CD45^+$ population [1].

Whole Blood Functional Tests: PFA-100 Closure Time (CT) and Whole Blood Coagulation Time

The PFA-100 CT was measured according to the gold standard guidelines using the collagen and ADP cartridge (CADP) [6]. Briefly, citrated blood was aspirated through a capillary in the CADP followed by platelet adhesion, activation and aggregate formation leading to occlusion of the central aperture and cessation of blood flow. Results are reported as CT in seconds (s), with values >300 s reported as non-closure [6].

Whole blood coagulation time was assessed by a one-stage clotting time, as described [3,7]. Briefly, 200 μL citrated blood was incubated for 2 h at 37 °C alone or with bacterial endotoxin (LPS) (100 ng/ml) or with tumor necrosis factor (TNF)-α (100 ng/ml). The optimal submaximal agonist concentration was selected on the basis of dose-response curves (not shown) in a preliminary study prior to the Moli-family recruitment. At the end of incubation, $CaCl_2$ (100 μL of 25 mM) was added to blood and the time of appearance of insoluble fibrin – clot formation was visually monitored by a trained technician and

recorded (coagulation time, in seconds; unstimulated: 399.47 ± 83.77 s; LPS stimulated: 255.40 ± 58.82 s; TNF-α stimulated: 352.83 ± 72.46 s; mean ± SD for all).

Supplementary Figure

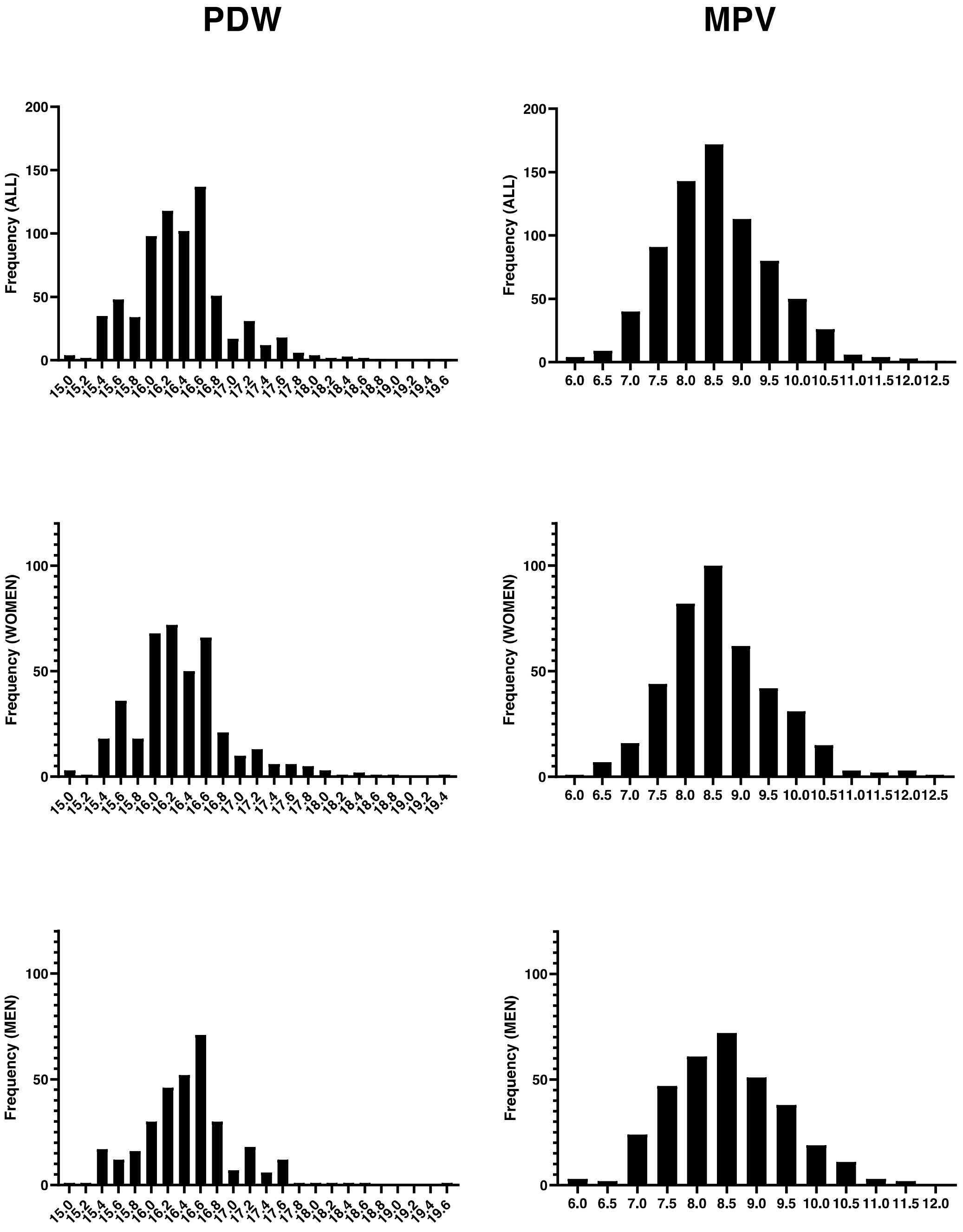

Supplementary Figure S1. PDW and MPV frequency distribution in the Moli-family cohort. Histogram representation of PDW and MPV distribution in the whole Moli-family cohort and in women and men separately. On X axes PDW or MPV fL distribution scale. Full descriptive for both PDW and MPV distribution is reported in Supplementary Table S7.

Supplementary Tables

Supplementary Table S1. Platelet indices, platelet activation/function markers and sex dependent differences of the Moli-family cohort

	ALL			WOMEN			MEN			p-value *
**Variable**	**N**	**Mean**	**SD**	**N**	**Mean**	**SD**	**N**	**Mean**	**SD**	
**PDW (fL)**	728	16.37	0.60	402	16.31	0.60	326	16.44	0.58	0.003
**MPV (fL)**	743	8.57	0.98	409	8.62	0.99	334	8.50	0.97	0.098
**Platelet count (10^9/L)**	743	251.07	62.84	409	263.42	63.77	334	235.96	58.31	1×10^{-6}
**Platelet P-selectin basal (%)**	728	2.72	3.67	403	2.73	3.84	325	2.70	3.46	0.913
**Platelet P-selectin ADP/Collagen (%)**	728	26.55	14.06	403	29.02	13.72	325	23.50	13.91	1×10^{-6}
**Platelet/monocyte aggregates basal (%)**	726	6.93	8.26	401	7.15	8.59	325	6.67	7.85	0.437
**Platelet/monocyte aggregates ADP/Collagen (%)**	727	17.49	20.51	402	20.62	22.94	325	13.63	16.26	4×10^{-6}
**Platelet/PMN aggregates basal (%)**	725	4.19	4.69	400	4.45	5.15	325	3.86	4.05	0.092
**Platelet/PMN aggregates ADP/Collagen (%)**	727	13.16	16.1	402	15.39	18.16	325	10.40	12.60	3×10^{-5}
**PFA-100 CT (seconds)**	728	97.66	27.98	402	98.43	29.67	326	96.71	25.74	0.410
**Coagulation time (seconds)**	728	399.47	83.77	402	398.55	85.42	326	400.63	81.79	0.739
**LPS stimulated coagulation time (seconds)**	728	255.4	58.82	402	252.54	59.86	326	258.98	57.39	0.142
**TNF-alpha stimulated coagulation time (seconds)**	726	352.83	72.46	401	351.58	75.12	325	354.39	69.08	0.604
**VWF (% VWF:Ag)**	725	118.26	48.21	400	119.54	48.89	325	116.64	47.37	0.421

*One-way ANOVA test

Supplementary Table S2. Pearson correlation matrix to test collinearity of PDW with the other platelet indices Plt and MPV

	Pearson correlation coefficient<br>Prob > |r| with H0: Rho=0<br>N								
	ALL			WOMEN			MEN		
	PDW	Plt	MPV	PDW	Plt	MPV	PDW	Plt	MPV
PDW	1	-0.199	0.032	1	-0.141	-0.003	1	-0.245	0.098
		<.0001*	0.382		0.0047*	0.945		<.0001*	0.078
	728	727	727	402	402	402	326	325	325
Plt	-0.199	1	-0.371	-0.141	1	-0.412	-0.242	1	-0.378
	<.0001*		<.0001*	0.005*		<.0001*	<.0001*		<.0001*
	727	743	742	402	409	409	325	333	333
MPV	0.032	-0.363	1	-0.003	-0.412	1	0.098	-0.378	1
	0.383	<.0001*		0.945	<.0001*		0.078	<.0001*	
	727	743	743	402	409	409	325	333	333

Significant p values are reported in red and highlighted by an asterisk

Supplementary Table S3. Plt association with P-selectin and coagulation assays in the Moli-family cohort

	**Plt**														
	**ALL@**					**WOMEN#**					**MEN#**				
	β	*SE*	*Probt*	*Lower*	*Upper*	*β*	*SE*	*Probt*	*Lower*	*Upper*	*β*	*SE*	*Probt*	*Lower*	*Upper*
**Platelet P-selectin basal**	0.046	0.037	0.207	-0.026	0.119	0.019	0.050	0.702	-0.079	0.117	0.098	0.052	0.062	-0.005	0.201
**Platelet P-selectin ADP/Collagen**	0.051	0.037	0.168	-0.022	0.124	0.075	0.053	0.160	-0.030	0.180	0.048	0.048	0.318	-0.047	0.144
**Platelet/monocyte aggregates basal**	0.131	0.035	**2.4E-04***	0.061	0.201	0.146	0.050	**0.003***	0.048	0.243	0.103	0.048	*0.034*	0.008	0.199
**Platelet/monocyte aggregates ADP/Collagen**	0.137	0.037	**2.1E-04***	0.065	0.210	0.163	0.050	**0.0005***	0.071	0.255	0.062	0.060	0.306	-0.057	0.181
**Platelet/PMN aggregates basal**	0.122	0.035	**4.9E-04***	0.053	0.190	0.110	0.046	*0.018*	0.019	0.201	0.159	0.052	**0.002***	0.057	0.262
**Platelet /PMN aggregates ADP/Collagen**	0.105	0.036	**0.003***	0.035	0.176	0.117	0.046	*0.011*	0.027	0.207	0.069	0.060	0.247	-0.048	0.187
**PFA-100 CT**	-0.003	0.001	*0.022*	-0.005	-0.0004	-0.003	0.002	0.106	-0.006	0.0006	-0.004	0.002	*0.030*	-0.007	-0.0004
**Coagulation time**	-0.609	0.193	**0.002***	-0.989	-0.229	-0.736	0.269	*0.007*	-1.265	-0.207	-0.414	0.264	0.118	-0.934	0.105
**LPS stimulated coagulation time**	-0.509	0.171	**0.003***	-0.844	-0.174	-0.584	0.244	*0.017*	-1,064	-0.105	-0.533	0.228	*0.020*	-0.982	-0.083
**TNF-alpha stimulated coagulation time**	-0.516	0.180	**0.004***	-0.870	-0.162	-0.457	0.247	0.065	-0.943	0.029	-0.677	0.255	*0.008*	-1.178	-0.175
**VWF**	-0.027	0.097	0.783	-0.216	0.163	-0.050	0.137	0.715	-0.320	0.220	0.103	0.131	0.434	-0.156	0.361

@Model adjusted by age and sex as fixed effect, family structure as random effect

#Model adjusted by age as fixed effect, family structure as random effect

Significant p values (< 0.007) are reported in red and highlighted by an asterisk, nominally significant p values (< 0.05) are reported in italics

Supplemental Table S4. MPV association with platelet activation, coagulation and inflammation related variables (Model adjusted by Plt)

	**MPV**														
	**ALL@**					**WOMEN#**					**MEN#**				
	β	*SE*	*Probt*	*Lower*	*Upper*	*β*	*SE*	*Probt*	*Lower*	*Upper*	*β*	*SE*	*Probt*	*Lower*	*Upper*
**Platelet P-selectin basal**	0.004	0.035	0.913	-0.065	0.073	0.016	0.043	0.703	-0.069	0.101	-0.050	0.059	0.399	-0.165	0.066
**Platelet P-selectin ADP/Collagen**	0.027	0.035	0.453	-0.043	0.096	-0.045	0.047	0.337	-0.136	0.047	0.082	0.054	0.131	-0.024	0.188
**Platelet/monocyte aggregates basal**	-0.031	0.034	0.365	-0.098	0.036	-0.019	0.044	0.658	-0.105	0.067	-0.038	0.055	0.488	-0.146	0.070
**Platelet/monocyte aggregates ADP/Collagen**	-0.014	0.036	0.700	-0.084	0.056	-0.060	0.045	0.149	-0.142	0.022	0.122	0.067	0.071	-0.011	0.255
**Platelet/PMN aggregates basal**	0.019	0.034	0.568	-0.047	0.085	0.039	0.041	0.340	-0.041	0.119	-0.030	0.060	0.620	-0.148	0.088
**Platelet /PMN aggregates ADP/Collagen**	0.031	0.034	0.366	-0.037	0.099	-0.003	0.040	0.940	-0.082	0.076	0.127	0.067	0.057	-0.004	0.258
**PFA-100 CT**	0.0002	0.001	0.858	-0.002	0.002	0.001	0.001	0.464	-0.002	0.004	-0.001	0.002	0.740	-0.005	0.003
**Coagulation time**	-0.094	0.187	0.615	-0.463	0.274	0.200	0.237	0.399	-0.266	0.665	-0.412	0.295	0.164	-0.994	0.169
**LPS stimulated coagulation time**	-0.348	0.164	*0.034*	-0.670	-0.026	-0.466	0.212	*0.029*	-0.884	-0.049	-0.139	0.258	0.591	-0.647	0.370
**TNF-alpha stimulated coagulation time**	-0.045	0.174	0.793	-0.387	0.296	0.072	0.215	0.737	-0.351	0.496	-0.197	0.290	0.497	-0.768	0.374
**VWF**	-0.100	0.091	0.271	-0.278	0.078	-0.006	0.118	0.960	-0.238	0.227	-0.252	0.144	0.081	-0.535	0.031
**Soluble P-selectin**	0.109	0.046	*0.018*	0.019	0.199	0.124	0.064	0.055	-0.003	0.251	0.117	0.064	0.071	-0.010	0.244
**CRP**	0.044	0.034	0.188	-0.022	0.110	0.039	0.045	0.386	-0.049	0.127	0.038	0.053	0.469	-0.066	0.143

@Model adjusted by age, sex and Plt as fixed effect, family structure as random effect

#Model adjusted by age and Plt as fixed effect, family structure as random effect

Nominally significant p values (< 0.05) are reported in italics

Supplementary Table S5. PDW association with platelet activation and coagulation variables (Model unadjusted by Plt)

	PDW														
	ALL@					WOMEN $					MEN $				
	β	*SE*	*Probt*	*Lower*	*Upper*	*β*	*SE*	*Probt*	*Lower*	*Upper*	*β*	*SE*	*Probt*	*Lower*	*Upper*
**Platelet P-selectin basal**	-0.147	0.037	9.4E-05*	-0.221	-0.074	-0.129	0.048	*0.008*	-0.224	-0.034	-0.177	0.059	0.003*	-0.293	-0.061
**Platelet P-selectin ADP/Collagen**	-0.008	0.039	0.845	-0.084	0.069	-0.028	0.053	0.596	-0.132	0.076	0.021	0.057	0.714	-0.091	0.133
**Platelet/monocyte aggregates basal**	-0.165	0.036	6.8E-06*	-0.237	-0.094	-0.168	0.048	5.9E-04*	-0.263	-0.073	-0.155	0.055	0.006*	-0.264	-0.046
**Platelet/monocyte aggregates ADP/Collagen**	-0.110	0.038	0.004*	-0.185	-0.034	-0.149	0.046	0.001*	-0.240	-0.059	-0.006	0.069	0.930	-0.143	0.131
**Platelet/PMN aggregates basal**	-0.149	0.036	3.7E-05*	-0.219	-0.079	-0.129	0.045	0.005*	-0.218	-0.039	-0.205	0.060	7.3E-04*	-0.323	-0.087
**Platelet /PMN aggregates ADP/Collagen**	-0.067	0.037	0.072	-0.140	0.006	-0.119	0.045	*0.008*	-0.207	-0.031	0.046	0.069	0.501	-0.089	0.181
**PFA-100 CT**	0.005	0.001	3.2E-05*	0.003	0.008	0.006	0.002	4.1E-04*	0.003	0.009	0.006	0.002	*0.007*	0.001	0.010
**Coagulation time**	0.473	0.201	*0.019*	0.077	0.868	0.328	0.265	0.218	-0.194	0.850	0.818	0.299	*0.007*	0.229	1.408
**LPS stimulated coagulation time**	0.744	0.175	2.4E-05*	0.400	1.087	0.687	0.238	0.004*	0.219	1.155	1.048	0.253	4.7E-05*	0.551	1.546
**TNF-alpha stimulated coagulation time**	0.670	0.185	3.3E-04*	0.306	1.035	0.636	0.241	*0.009*	0.162	1.111	0.883	0.288	0.002*	0.316	1.450
**VWF**	-0.298	0.099	0.003*	-0.493	-0.103	-0.457	0.133	7.4E-04*	-0.713	-0.191	-0.139	0.151	0.358	-0.437	0.159

@Model adjusted by age and sex as fixed effect, family structure as random effect

$ Model adjusted by age as fixed effect, family structure as random effect

Significant p values (< 0.007) are reported in red and highlighted by an asterisk, nominally significant p values (< 0.05) are reported in italics

Supplementary Table S6. MPV association with platelet activation and coagulation variables (Model unadjusted by Plt)

	MPV														
	ALL@					WOMEN $					MEN $				
	β	*SE*	*Probt*	*Lower*	*Upper*	*β*	*SE*	*Probt*	*Lower*	*Upper*	*β*	*SE*	*Probt*	*Lower*	*Upper*
**Platelet P-selectin basal**	-0.014	0.038	0.721	-0.088	0.061	0.012	0.047	0.799	-0.081	0.105	-0.088	0.062	0.159	-0.210	0.034
**Platelet P-selectin ADP/Collagen**	0.007	0.038	0.857	-0.069	0.082	-0.071	0.051	0.162	-0.172	0.029	0.064	0.057	0.265	-0.049	0.177
**Platelet/monocyte aggregates basal**	-0.083	0.037	*0.024*	-0.155	-0.011	-0.072	0.047	0.127	-0.165	0.021	-0.080	0.058	0.169	-0.193	0.034
**Platelet/monocyte aggregates ADP/Collagen**	-0.069	0.038	0.072	-0.145	0.006	-0.123	0.045	0.006*	-0.211	-0.035	0.100	0.071	0.165	-0.041	0.240
**Platelet/PMN aggregates basal**	-0.029	0.036	0.422	-0.100	0.042	-0.001	0.044	0.979	-0.088	0.086	-0.092	0.062	0.143	-0.215	0.031
**Platelet /PMN aggregates ADP/Collagen**	-0.011	0.037	0.760	-0.085	0.062	-0.051	0.043	0.254	-0.135	0.036	0.103	0.071	0.148	-0.037	0.242
**PFA-100 CT**	0.001	0.001	0.297	-0.001	0.004	0.002	0.002	0.194	-0.001	0.005	0.001	0.002	0.659	-0.003	0.005
**Coagulation time**	0.142	0.202	0.481	-0.254	0.538	0.481	0.256	0.061	-0.023	0.986	-0.271	0.313	0.388	-0.888	0.347
**LPS stimulated coagulation time**	-0.150	0.177	0.396	-0.498	0.197	-0.241	0.231	0.301	-0.695	0.216	0.070	0.271	0.797	-0.464	0.604
**TNF-alpha stimulated coagulation time**	0.153	0.187	0.412	-0.214	0.520	0.248	0.234	0.290	-0.213	0.709	0.054	0.304	0.861	-0.546	0.653
**VWF**	-0.085	0.099	0.392	-0.279	0.109	0.017	0.131	0.898	-0.239	0.272	-0.294	0.153	0.056	-0.595	0.007

@Model adjusted by age and sex as fixed effect, family structure as random effect

$ Model adjusted by age as fixed effect, family structure as random effect

Significant p values (< 0.007) are reported in red and highlighted by an asterisk, nominally significant p values (< 0.05) are reported in italics

Supplementary Table S7. PDW and MPV descriptive in the Moli-family cohort

	PDW (fL)			MPV (fL)		
	ALL	WOMEN	MEN	ALL	WOMEN	MEN
**Number of subjects**	727	402	325	742	409	333
**Minimum**	15.1	15.1	15.1	6.0	6.0	6.1
**25% Percentile**	16.0	15.9	16.1	7.9	8.0	7.8
**Median**	16.3	16.2	16.4	8.4	8.5	8.4
**75% Percentile**	16.7	16.6	16.7	9.2	9.2	9.15
**Maximum**	19.5	19.3	19.5	12.4	12.4	11.6
**Range**	4.4	4.2	4.4	6.4	6.4	5.5
**10% Percentile**	15.7	15.6	15.8	7.4	7.6	7.3
**90% Percentile**	17.1	17.0	17.2	9.9	9.9	9.8
**95% CI Lower limit**	16.2	16.1	16.3	8.4	8.4	8.3
**95% CI Upper limit**	16.4	16.3	16.5	8.6	8.6	8.5
**Mean**	16.4	16.3	16.4	8.6	8.6	8.5
**Std. Deviation**	0.6	0.6	0.6	1.0	1.0	1.0
**Std. Error of Mean**	0.02	0.03	0.03	0.04	0.05	0.05
**Skewness**	1.05	1.2	0.9	0.5	0.6	0.3
**Kurtosis**	2.5	2.8	2.6	0.6	1.01	-0.03

Moli-Family Study Investigators

The enrollment phase of the Moli-family Study was conducted at the Research Laboratories of the Catholic University in Campobasso (Italy) between 2005–2007. From 2013, the study is being conducted at the Department of Epidemiology and Prevention of the IRCCS Neuromed, Pozzilli, Italy.

Principal Investigator: Licia Iacoviello (IRCCS Neuromed, Pozzilli, and University of Insubria, Varese, Italy).

Scientific Committee: Chiara Cerletti, Maria Benedetta Donati, Giovanni de Gaetano (IRCCS Neuromed, Pozzilli, Italy), Augusto Di Castelnuovo (Mediterranea Cardiocentro, Napoli, Italy), and Branislav Vohnout (Slovak Medical University, Bratislava, Slovakia).

Data management and analysis: Augusto di Castelnuovo (Mediterranea Cardiocentro, Napoli, Italy), Francesco Gianfagna (Mediterranea Cardiocentro, Napoli, Italy, and University of Insubria, Varese, Italy), Simona Costanzo, Alessandro Gialluisi, Sabatino Orlandi (IRCCS Neuromed, Pozzilli, Italy), and Marco Olivieri (Associazione Cuore Sano, ONLUS, Campobasso),

Biobank and centralized laboratory: Amalia De Curtis (IRCCS Neuromed, Pozzilli, Italy) and Sara Magnacca (Mediterranea Cardiocentro, Napoli, Italy).

Genetic analyses: Benedetta Izzi, Annalisa Marotta, Fabrizia Noro, Roberta Parisi, and Alfonsina Tirozzi (IRCCS Neuromed, Pozzilli, Italy).

References

1. Izzi, B.; Pampuch, A.; Costanzo, S.; Vohnout, B.; Iacoviello, L.; Cerletti, C.; de Gaetano, G. Determinants of platelet conjugate formation with polymorphonuclear leukocytes or monocytes in whole blood. *Thromb Haemost* **2007**, *98*, 1276-1284.
2. Izzi, B.; Gianfagna, F.; Yang, W.Y.; Cludts, K.; De Curtis, A.; Verhamme, P.; Di Castelnuovo, A.; Cerletti, C.; Donati, M.B.; de Gaetano, G.; et al. Variation of PEAR1 DNA methylation influences platelet and leukocyte function. *Clin Epigenetics* **2019**, *11*, 151, doi:10.1186/s13148-019-0744-8.
3. Noro, F.; Gianfagna, F.; Gialluisi, A.; De Curtis, A.; Di Castelnuovo, A.; Napoleone, E.; Cerletti, C.; Donati, M.B.; de Gaetano, G.; Hoylaerts, M.F.; et al. ZBTB12 DNA methylation is associated with coagulation- and inflammation-related blood cell parameters: findings from the Moli-family cohort. *Clin Epigenetics* **2019**, *11*, 74, doi:10.1186/s13148-019-0665-6.
4. Di Castelnuovo, A.; de Curtis, A.; Costanzo, S.; Persichillo, M.; Olivieri, M.; Zito, F.; Donati, M.B.; de Gaetano, G.; Iacoviello, L.; Investigators, M.-S.P. Association of D-dimer levels with all-cause mortality in a healthy adult population: findings from the MOLI-SANI study. *Haematologica* **2013**, *98*, 1476-1480, doi:10.3324/haematol.2012.083410.
5. Gianfagna, F.; Tamburrelli, C.; Vohnout, B.; Crescente, M.; Izzi, B.; Pampuch, A.; De Curtis, A.; Di Castelnuovo, A.; Cutrone, A.; Napoleone, E.; et al. Heritability, genetic correlation and linkage to the 9p21.3 region of mixed platelet-leukocyte conjugates in families with and without early myocardial infarction. *Nutr Metab Cardiovasc Dis* **2013**, *23*, 684-692, doi:10.1016/j.numecd.2012.02.008.
6. Hayward, C.P.; Harrison, P.; Cattaneo, M.; Ortel, T.L.; Rao, A.K.; Platelet Physiology Subcommittee of the, S.; Standardization Committee of the International Society on, T.; Haemostasis. Platelet function analyzer (PFA)-100 closure time in the evaluation of platelet disorders and platelet function. *J Thromb Haemost* **2006**, *4*, 312-319, doi:10.1111/j.1538-7836.2006.01771.x.
7. Napoleone, E.; di Santo, A.; Peri, G.; Mantovani, A.; de Gaetano, G.; Donati, M.B.; Lorenzet, R. The long pentraxin PTX3 up-regulates tissue factor in activated monocytes: another link between inflammation and clotting activation. *J Leukoc Biol* **2004**, *76*, 203-209, doi:10.1189/jlb.1003528.
